# Human Activity in Antarctica: Effects on Metallic Trace Elements (MTEs) in Plants and Soils

**DOI:** 10.3390/plants10122593

**Published:** 2021-11-26

**Authors:** Jaime Tapia, Marco Molina-Montenegro, Camila Sandoval, Natalia Rivas, Jessica Espinoza, Silvia Basualto, Pablo Fierro, Luis Vargas-Chacoff

**Affiliations:** 1 Institute of Natural Resources Chemistry, University of Talca, Talca 3460000, Chile; j.espinoza@utalca.cl; 2 Institute of Biological Sciences, University of Talca, Talca 3460000, Chile; marco.molina@utalca.cl (M.M.-M.); camisando28@gmail.com (C.S.); natalirivasj@gmail.com (N.R.); 3 Centro de Estudios Avanzados en Zonas Áridas, CEAZA, Universidad Católica del Norte, Coquimbo 1780000, Chile; 4 EULA Center, University of Concepción, Concepción 4030000, Chile; sbasualt@udec.cl; 5 Institute of Marine Science and Limnology, University Austral of Chile, Valdivia 5090000, Chile; 6 Centro Fondap de Investigación de Altas Latitudes, FONDAP-IDEAL Center, University Austral of Chile, Valdivia 5090000, Chile; 7 Millennium Institute Biodiversity of Antarctic and Subantarctic Ecosystems, BASE, University Austral of Chile, Valdivia 5090000, Chile

**Keywords:** bioaccumulation, biomonitoring, *Colobanthus quitensis*, metallic trace elements, soil pollution

## Abstract

*Colobanthus quitensis* (Kunt) is one of the two vascular plant species present in Antarctica and develops under severe environmental conditions, being found in both pristine and human-threatened environments. We determined the Cd, Cr, Cu, Mn, Ni, Pb, and Zn levels in *C. quitensis* roots, leaves, and soils of origin using flame atomic absorption spectroscopy. In January 2017, we collected samples from four geographical zones on the longitudinal gradient along which *C. quitensis* is distributed, starting from Punta Arenas (PAR) at the extreme south of mainland Chile and moving southwards to the Antarctic territory from King George Island (KGI) to Hannah Point Peninsula (PHA) and finally Lagotellerie Island (LAT). We used certified reference material to validate the plant tissues and soil samples we collected. The highest concentrations of metals that we measured in the soils and in the *C. quitensis* roots and leaves were in samples we collected at the KGI station, the zone with the greatest human activity. The lowest concentrations we measured were at the LAT station, an island with little human intervention and scarce fauna. The mean concentrations of metals in the roots and leaves of *C. quitensis* followed a similar order at all sampling locations: Mn > Zn > Cu > Ni > Pb > Cr > Cd. In contrast, in soil, they followed the following order: Mn > Zn > Cu > Cr > Pb > Ni > Cd. The concentration levels obtained for the different metals in the soil and plants tissue samples in this region of Antarctica indicated that the area was non-polluted. However, the metallic trace element (MTE) concentrations may be at an early stage of contamination, as described in other areas of the Antarctic, being a new threat to this continent.

## 1. Introduction

The Antarctic continent is considered the last pristine continent; however, increasing human activity and permanent installation of research stations threaten the Antarctic environment [1]. Pollution in the Antarctic is related to specific contaminants, such as metallic trace elements (MTEs), with high levels found close to research stations [2,3]. MTEs enter the environment naturally through the erosion of geological matrices and atmospheric deposition; however, high levels of MTEs in the terrestrial environment are most often a result of human activity [4,5]. These elements are not naturally biodegradable; they are stable and can be transferred over considerable distances, allowing for bio-accumulation in different animals and plants [6,7,8]. Certain metals such as iron, zinc, copper, manganese, etc. are essential trace elements for maintaining the correct metabolism in living creatures. However, when they exceed specific concentrations, they become toxic [9].

During the last decades, there has been growing interest in measuring concentrations of MTEs within different parts of the environment, such as air, water, and soil. MTE concentrations in other animals and plants can be bioindicators of MTE concentrations in specific habitats [10,11,12,13,14,15]. The absorption of metals into plants depends on the mobility and chemistry of these elements in the soil surrounding the plant’s roots, and some chemical forms have greater bio-availability than others [16]. Metal atoms are present in the ground in different compounds; for example, Mn can be present as an oxide and a hydroxide, forming molecules with other metals. Mn is an essential element in plant nutrition, differing greatly by normal contents between species (30–500 mg/kg) [17]. It is required to activate certain enzymes in the plant metabolism [18]. Ni appears to be bound to organic forms, principally forming readily soluble chelates [19], and is also an essential element for plant metabolism but only in optimal concentrations, i.e., below the critical plant concentration range of 10–100 mg/kg [20]. Other metals needed by plants as micronutrients include boron, cobalt, chromium, copper, molybdenum, iron, selenium, and zinc; however, above a certain threshold, they all become toxic. In soils, for example, according to the Council Directive [21], certain concentrations above a threshold have harmful effects on the environment: for Cd, 3.0 mg/kg; for Cu, 140 mg/kg; for Ni, 75 mg/kg; and for Pb and Zn, 300 mg/kg. Cadmium, mercury, lead, tin, and bismuth are the main elements that can become highly toxic to living creatures because they have a strong tendency both to bio-accumulate and to be bio-magnified [22,23].

We determined the presence of certain MTEs on a geographical transect from the south of mainland Chile to Antarctica, using *Colobanthus quitensis* (Kunt), also known as “Antarctic carnation”, as a bioindicator of these metals. *C. quitensis* is one of two vascular plant species (together with *Deschampsia antarctica* Desv) present in the vegetation of Antarctica [24], where environmental conditions, such as low temperature, high solar radiation, and low availability of water and nutrients in the soil [25,26], are highly stressful for plant development [27]. The physiological, ecological, reproductive, and taxonomic characteristics of these plants are of great interest because of the environmental stress they can endure [28,29,30,31,32,33,34].

To determine the concentrations of metals included in the present study, we selected four geographical stations along a longitudinal gradient that followed the distribution of *Colobanthus quitensis* from the south of mainland Chile to Antarctica. We analyzed metal concentrations in the plants by taking representative samples of the leaves, roots, and soil of origin at each sampling station. We measured the Mn, Cu, Ni, and Zn levels because they are necessary for plant growth in optimal concentrations and Pb, Cd, and Cr because of their known toxic characteristics. We hypothesized that plants inhabiting soils close to human activities have higher MTE concentrations than plants inhabiting soils with low human impact. We compared the concentrations of these metals with other studies in different plant species. Our results increase our understanding of the ecological effects on one of the two vascular plants associated with human activities on the Antarctic continent and contribute to the development of *C. quitensis* as a bioindicator of MTEs.

## 2. Results

Table A1 and Table A2 in Appendix A show the results of the Cd, Cu, Cr, Mn, Ni, Pb, and Zn measurements in the BIMEP-432 and MESS–1 reference materials, corresponding to plant tissues and soils, respectively. The relative errors for plant tissue ranged between −8.5% (Cd) and 7.8% (Pb). The relative errors for soils samples ranged between −2.6% (Cr) and 5.1% (Cd).

**Figure 1:**
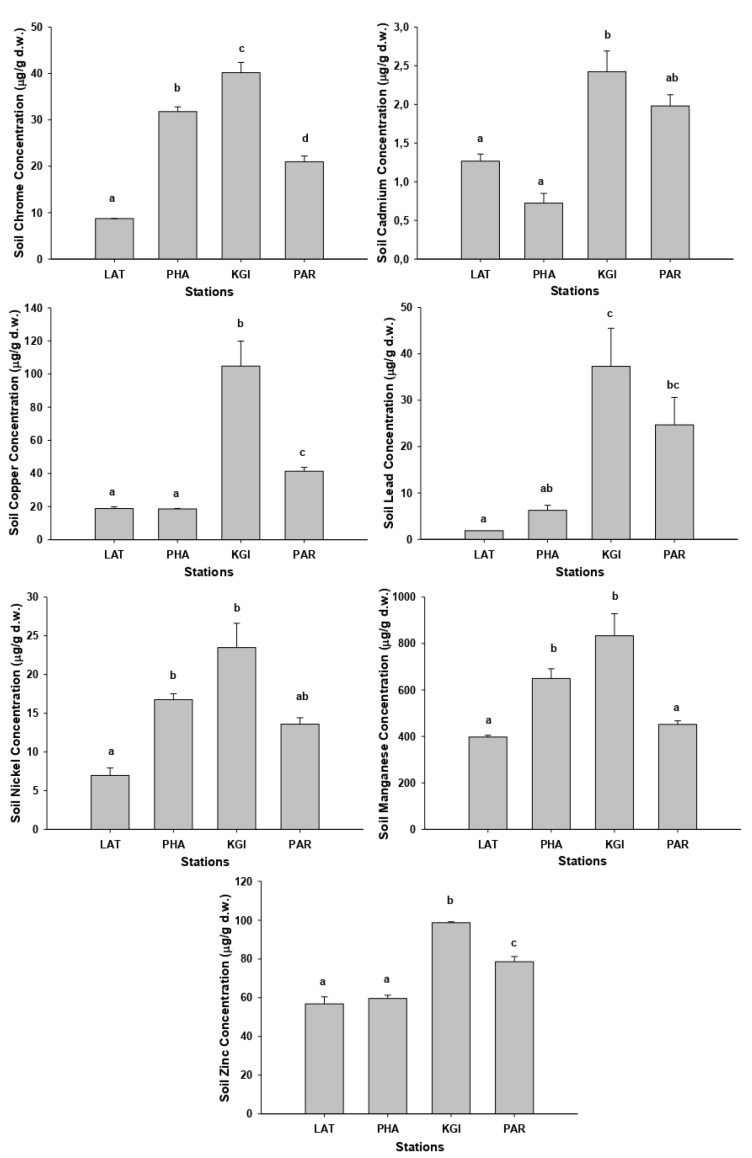
Graphic representation of the concentrations (µg/g dry weight) of Cd, Cu, Cr, Mn, Ni, Pb, and Zn in the soils of the species *Colobanthus quitensis* collected at the stations Lagotellerie Island (LAT), Hannah Point Peninsula (PHA), King George Island (KGI), and Punta Arenas (PAR). Different letters indicated statistical differences (*p* < 0.05).

The mean concentrations of the metals studied in all soil samples exhibited the following pattern: Mn (mean 582.9 mg/kg) > Zn (mean 70 mg/kg) > Cu (mean 45.9 mg/kg) > Cr (mean 25.4 mg/kg) > Pb (mean 17.5 mg/kg) > Ni (mean 15.2 mg/kg) > Cd (mean 1.5 mg/kg). The highest mean concentrations of the most metals in soils were recorded at KGI station (except Cd, in which the highest mean concentration was founded in PAR station), whereas the lowest concentrations for the many metals were recorded in the LAT station followed by PHA station (Appendix B Table A3). The metal concentrations recorded at KGI station were significantly different (*p* < 0.05) from those recorded at the LAT, PHA, and PAR stations (Figure 1).

**Figure 2:**
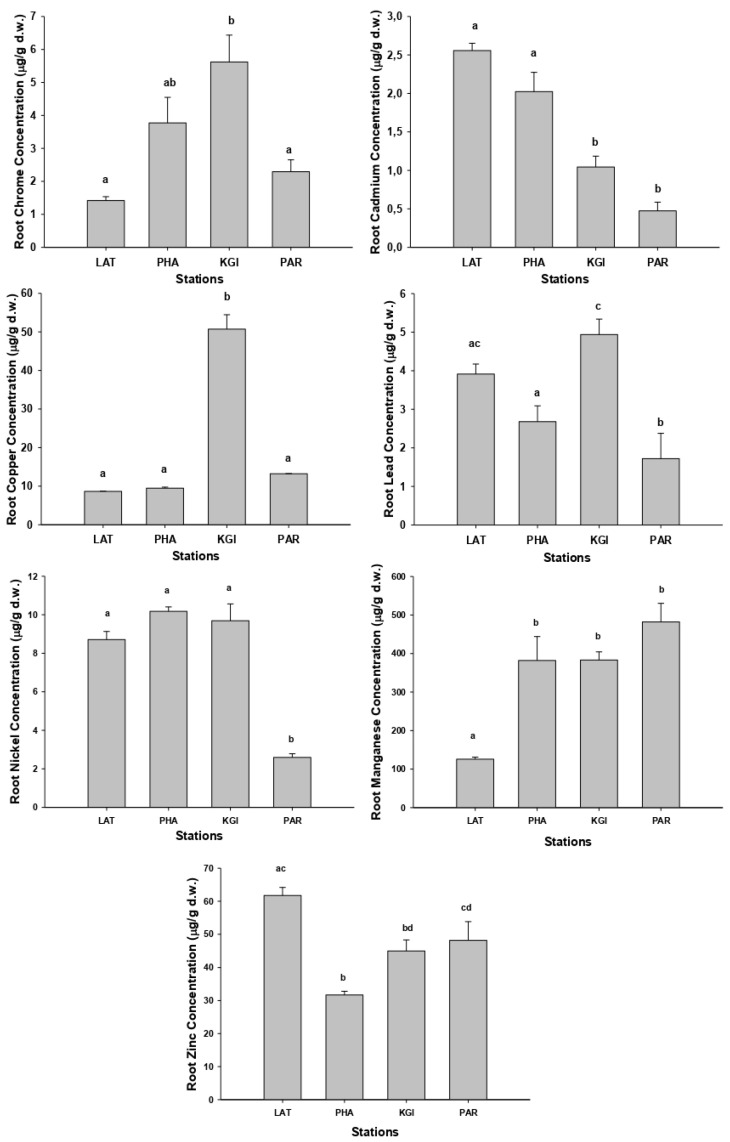
Graphic representation of the concentrations (µg/g dry weight) of Cd, Cu, Cr, Mn, Ni, Pb, and Zn in the roots of species *Colobanthus quitensis* collected at the stations: Lagotellerie Island (LAT), Hannah Point Peninsula (PHA), King George Island (KGI), and Punta Arenas (PAR). Different letters indicated statistical differences (*p* < 0.05).

**Figure 3:**
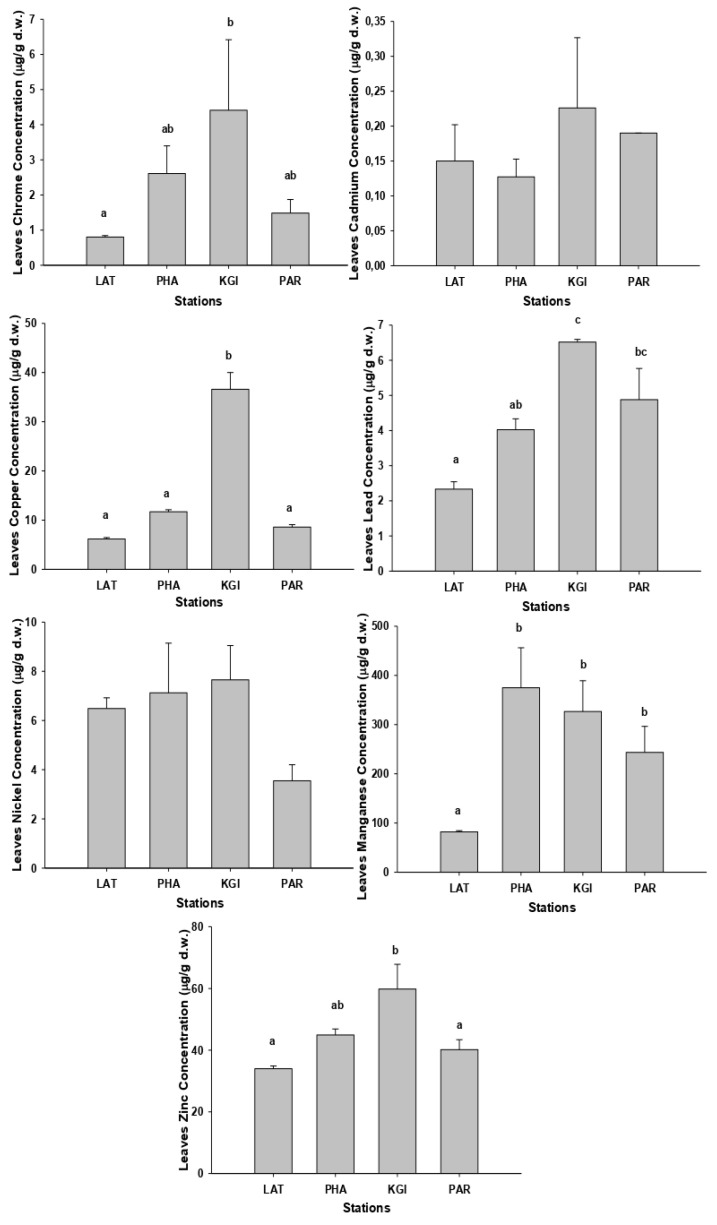
Graphic representation of the concentrations (µg/g dry weight) of Cd, Cu, Cr, Mn, Ni, Pb, and Zn in the leaves of species *Colobanthus quitensis* collected at the stations: Lagotellerie Island (LAT), Hannah Point Peninsula (PHA), King George Island (KGI), and Punta Arenas (PAR). Different letters indicated statistical differences (*p* < 0.05).

As with the soil samples, the highest concentrations of metals in roots and leaves were recorded in the samples collected at the KGI station (Figure 2 and Figure 3) and the mean concentrations for the different metals in roots and leaves showed the same order of concentrations at all stations: Mn (mean 343.2 mg/kg-roots, 256.7 mg/kg-leaves) > Zn (mean 46.7 mg/kg-roots, 44.7 mg/kg-leaves) > Cu (mean 20.9 mg/kg-roots, 15.8 mg/kg-leaves) > Ni (mean 7.8 mg/kg-roots, 6.2 mg/kg-leaves) > Pb (mean 3.4 mg/kg-roots, 4.4 mg/kg-leaves) > Cr (mean 3.3 mg/kg-roots, 2.3 mg/kg-leaves) > Cd (mean 1.5 mg/kg-roots, 0.2mg/kg-leaves). The mean concentrations of the metals were higher in the roots than the leaves, except for Pb (Appendix B Table A4 and Table A5).

The metal concentrations in the roots of *Colobanthus quitensis* (Figure 2) show that samples collected at KGI station presented the highest concentrations of chromium, copper, and lead, with significant differences compared with the other stations (*p* < 0.05). The samples collected in the LAT station contained the highest concentrations of cadmium and zinc. In contrast, we found the highest concentrations of manganese in the roots of *C. quitensis* from the PAR, PHA, and KGI stations, but there were no significant differences between them (*p* > 0.05). We recorded the highest nickel concentrations in the root samples from the LAT, PAH, and KGI stations, with no significant differences between them (*p* > 0.05).

We detected the highest concentrations of chromium in the leaves of *C. quitensis*, (Figure 3) at KGI station, but the difference from the PHA and PAR station values was not significant (*p* > 0.05). The samples collected in the KGI station presented significantly (*p* < 0.05) higher copper concentrations than the other sites. We recorded the highest concentrations of cadmium and zinc at KGI station; however, they were not significantly different from other stations (*p* > 0.05). The highest concentrations of Pb were recorded in leaf samples from the KGI and PAR stations but were not significantly different from the other sites (*p* > 0.05). The highest concentrations of manganese were present in the leaves from the PHA station, but the differences for the KGI and PAR stations were also not statistically significant (*p* > 0.05).

**Figure 4:**
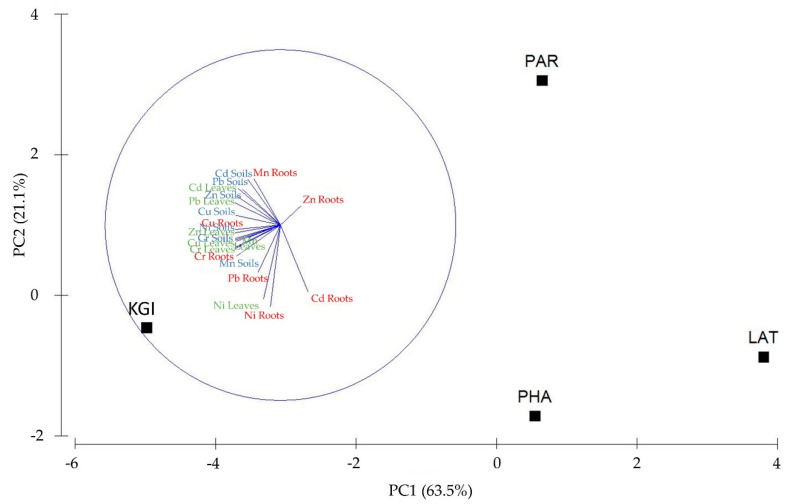
Principal component analysis (PCA) of MTEs in the leaves (green color); roots (red color) of *Colobanthus quitensis*; and soils (blue color) from Lagotellerie Island (LAT), Hannah Point Peninsula (PHA), King George Island (KGI), and Punta Arenas (PAR).

PCA analysis confirmed that the most robust relationship between high levels of MTEs in the leaves, roots, and soils, and a specific sampling site was at the KGI site. PCA axis 1 explained 63.5%, and axis 2 explained 21.1% of the total variance in the MTE data. The first axis was mainly related to MTEs in the leaves and soils associated with KGI station. In contrast, the second axis was primarily related to roots associated with KGI and PHA stations (Figure 4).

## 3. Discussion

The soils of the Antarctic Peninsula and the roots and leaves of Antarctic carnation had higher MTE concentrations at King George Island Station, characterized by a strong human influence. Increased MTE concentrations in Antarctica have previously been reported in sediments and in terrestrial and aquatic organisms and are related to human activity [3,35,36].

The MTE concentrations recorded in the soils in this study were compared with those by other authors in different areas from King George Island and for other Antarctic regions (Table 1) [37,38]. Although we recorded the highest concentrations of metals in soils at KGI station in our study, they were lower than those reported in soil from Fildes Peninsula and other Antarctic islands. Moreover, the Cr, Cu, Mn, Ni, Pb, and Zn concentrations were within the limits permitted by the Canadian CCME standards [39,40,41], the standards of the European Union Directive 86/278/EEC national legislation [21], and the World Range for non-polluted soils [42]. Only the average concentration and the highest Cd values were higher than those in other studies, and the Cd and Cu average concentration values were above that of the World Range for non-polluted soils recommendations. Therefore, our results suggest that soils in this region of Antarctica remain non-polluted soils; however, they may be at an early stage of contamination, as in other areas of the Antarctic [3].

Romaniuk et al. [43] have already reported that contaminated soils from King George Island have high concentrations of MTEs. On the other hand, LAT station was the southernmost point of Antarctica from which we took samples and had the lowest concentrations of metals in soil (Lagotellerie Island). This area had very little human intervention, appeared pristine, and had little fauna, suggesting that the levels of metals we measured may represent those that occur naturally [33]. In samples from coastal lagoons of south Chile, Mn and Zn concentrations are the metals with the highest concentrations in soil and occur naturally [44].

To our knowledge, this is the second study that addresses the MTEs concentration in *Colobanthus quitensis* in Antarctica. The first was performed by Wojtuń et al. [45], with samples collected in 1988 on King George Island, close to our study area. However, it is impossible to compare their results with ours because we analyzed the metals in *C. quitensis* in conjunction with the Antarctic hair grass *Deschampsia antarctica*. Despite this, the mean concentrations we recorded in leaves and roots for the different metals are similar to those published by other authors in various plants (Table 2) [14,46,47]. Information about the vascular plants present in Antarctica is limited. Dos Santos et al. [48] measured the concentrations of three MTEs in samples of *Deschampsia antarctica* collected from Admiralty Bay (King George Island): 0.06 Hg, 610 Fe, and 44.2 Zn (in mg/kg). Although only the Zn values could be compared with our samples, the mean concentrations were quite similar. The sensitivity of plant species to MTEs varies in different orders and families and, vascular plants are more tolerant than other taxa [49].

We found that the metals with the highest mean concentrations in the root and leaves samples of *Colobanthus quitensis* were Mn, Zn, Cu, and Ni. Mn is an essential natural element in plant nutrition and is required to activate some enzymes [18]. However, when it is present in the soil in high concentrations, passive absorption may occur, and the metal is transferred to other organs of the plant [16]. Zn is an essential element in plants, playing a part in different metabolic processes and is a component of various enzymes. Zn enters the plant as Zn^+2^ or in the form of organic complexes and chelates. Zn can accumulate in the canopies of plants when it is present at high levels [16]. Cu readily forms complexes with organic matter in the soil, and its bio-availability is likely to be associated with the presence of these compounds [50]. The mechanism by which plants absorb Cu is not yet well-defined. It has a relatively lower mobility in plants than other elements and remains in the roots and leaves until the plant ages [51,52]. Ni is essential for plant metabolism in optimal concentrations [18]. It is mobile within the plant and accumulates mainly in the leaves and seeds. Plants exposed to high concentrations of Ni develop chlorosis [53].

The metals with the lowest concentrations in the roots and leaves of *Colobanthus quitensis* were Cr, Pb, and Cd. Cr has three chemical forms: metallic chromium, chromite (trivalent Cr (III)), and chromate (hexavalent Cr (VI)), with the last one being the most common form found in nature. Chromate is the most bio-available form to plants when present in the soil, but it is extremely unstable and easily mobilized in both acidic and alkaline soils [54,55]. Cr is accumulated predominantly in plant root with very limited translocation to other parts of the plants, as shoots or leaves, inducing phytotoxicity by interfering plant growth, nutrient uptake, and photosynthesis [56]. According to our results, higher levels of Cr were recorded in roots than in leaves. The plant intake with high levels of Cr could be highly toxic and damaging to human health because of its carcinogenic and mutagenic effects with no nutritional value [57]. Pb in the environment is associated with human activities, for example, until the mid-1970s, lead was used as a fuel additive and as a component of paints in developed countries, whereas today, Pb presence is only possibly found in paints in developing countries, such as Chile. Therefore, its presence in soils and plant leaves can be attributed to atmospheric deposition due to the degradation of paints on exposed surfaces [43].

Although Pb is not known to play a role in plant metabolism, it is easily absorbed by plants and it is principally stored in the roots but is not present in the reproductive structures [58]. However, in high concentrations, Pb cause several toxicity symptoms in plants, such as chlorosis, stunted growth, blackening of the root system, nutrient uptake, water imbalance, and photosynthesis inhibition [59]. Cd is one of the most soluble and dangerous trace metals in the soil due to its high mobility and has harmful effects on plants at very low concentrations. High Cd concentrations in the soil inhibit the absorption of Fe in plants, and the most common symptoms of Cd toxicity in plants are atrophy and chlorosis. Cd enters the environment from tires and motor oil [60]. Therefore, its presence can be attributed to atmospheric deposition from vehicle exhaust emissions.

**Table 1:** Selected references of the MTE concentrations (mg/kg in dry weight) in soils. Values are mean ± standard deviation and ranges). Impacted soils are principally by scientific stations. Values ranges are according to the EUS (European Union Standard) and WRS (World Range for non-polluted soils), and mean values are according to CA (Canadian Standard).

Reference	Location	Sample	Cd	Cr	Cu	Mn	Ni	Pb	Zn
Present study (Mean)	Southern Chile and King George Island	Control/impacted soils	1.5 ± 0.5	25.4 ± 11.8	45.9 ± 35.2	582.9 ± 172.3	15.2 ± 5.9	17.5 ± 14.3	70.0 ± 12.0
Present study (Range)			1.98–0.72	40.17–8.73	104.8–18.59	833.39–397.29	23.48–6.95	37.3–1.87	84.8–56.8
De Lima Neto et al. [61]	Luis Risopatrón Refuge	Control soils	<0.2	52 ± 1	47.8 ± 0.4	453 ± 3	40.4 ± 0.5	7.3 ± 0.5	43.9 ± 0.2
		Impacted soils	<0.2	72 ± 2	107 ± 1	545 ± 10	37.9 ± 0.5	102 ± 0.5	148 ± 5
Lu et al. [37]	Fildes Peninsula	Impacted soils	0.17 ± 0.08	31.95 ± 13.82	122.30 ± 32.12	923 ± 244.60	14.30 ± 4.97	15.87 ± 13.49	58.69 ± 9.36
Abakumov et al. [62]	Fildes Peninsula	Impacted soils	0.44 ± 0.22	-	186.33 ± 36	720 ± 218.33	15.66 ± 5.50	-	134.67 ± 26.50
		Pristine soils	0.25 ± 0.13	-	65 ± 13	616.25 ± 183.75	11.22 ± 3.93	-	42.10 ± 8.48
Bueno et al. [38]	Artigas Base	Impacted soils	-	10.3 ± 8.1	52.2 ± 9.1	503.9 ± 81.6	5.2 ± 1.9	6.3 ± 3.8	61.3 ± 28.5
EUS (1)			1–3	-	50–140	-	30–75	50–300	150–300
CS (2)			10	64	63	-	45	140	200
WRS (3)			0.07–1.1	5–120	6–60	-	1–200	10–70	17–125
(1) *Council Directive 86/278/EEC* [21]									
(2) *CCME* [39,40,41]									
(3) *Frimpong and Koranteng* [42]									

**Table 2:** Selected references of MTE concentrations (mg/kg in dry weight) in vegetables. Values are mean ± standard deviation and ranges.

Reference	Location	Sample	Cd	Cr	Cu	Mn	Ni	Pb	Zn
Present study (Mean)	Southern Chile and King George Island	Roots (1)	1.5 ± 0.8	3.3 ± 1.6	20.9 ± 17.2	343.2 ± 131.9	7.8 ± 3.0	3.4 ± 1.3	46.7 ± 10.7
		Leaves (1)	0.2 ± 0.04	2.3 ± 1.4	15.8 ± 12.2	256.7 ± 111.3	6.2 ± 1.6	4.4 ± 1.5	44.7 ± 9.6
Dos Santos et al. [48]	King George Island	Plant (2)	-	-	-	-	-	-	44.2
Nematollahi et al. [47]	Iran	Roots (3)	0.9	3	241.5	27.3	1.7	13	23.6
		Leaves (3)	0.9	6.4	365.2	90.8	4.4	30.7	56.3
Kazienko et al. [14]	Poland	Plant (4)	0.7 ± 0.4	0.2 ± 0.1	8.0 ± 1.4	317 ± 101	6.6 ± 4.0	1.3 ± 0.5	31 ± 6.0
Tapia et al. [46]	Chile	Plant (5)	-	0.2–3.2	1.2–62.5	188.6–1345.1	-	-	7.5–55.9
(1) *Colobantus quitensis*									
(2) *Deschampsia antarctica*									
(3) *Artemisia sieberi*									
(4) *Athyrium distentifolium*									
(5) *Azorella spinace*									

## 4. Materials and Methods

### 4.1. Study Area

Punta Arenas city has a surface area of 37.6 km^2^ and has a population of 124,000 inhabitants, and it is approximately 1200 km from King George Island (KGI). Lagotellerie Island has a surface area of 1.58 km^2^ and is approximately 450 km from KGI, with the latter island having a surface area of 1400 km^2^. In the study, there are two major climates: (a) the cold steppe climate in Punta Arena city has short-fresh summers and not very cold winters, with the average temperature being 6.7 °C and the annual precipitation being <500 mm; (b) the subpolar maritime climate in King George and Lagotellerie Islands has a cold, moist, and maritime climate, with the average temperature being −2.4 °C and the annual precipitation being 500 mm [63,64].

**Figure 5:**
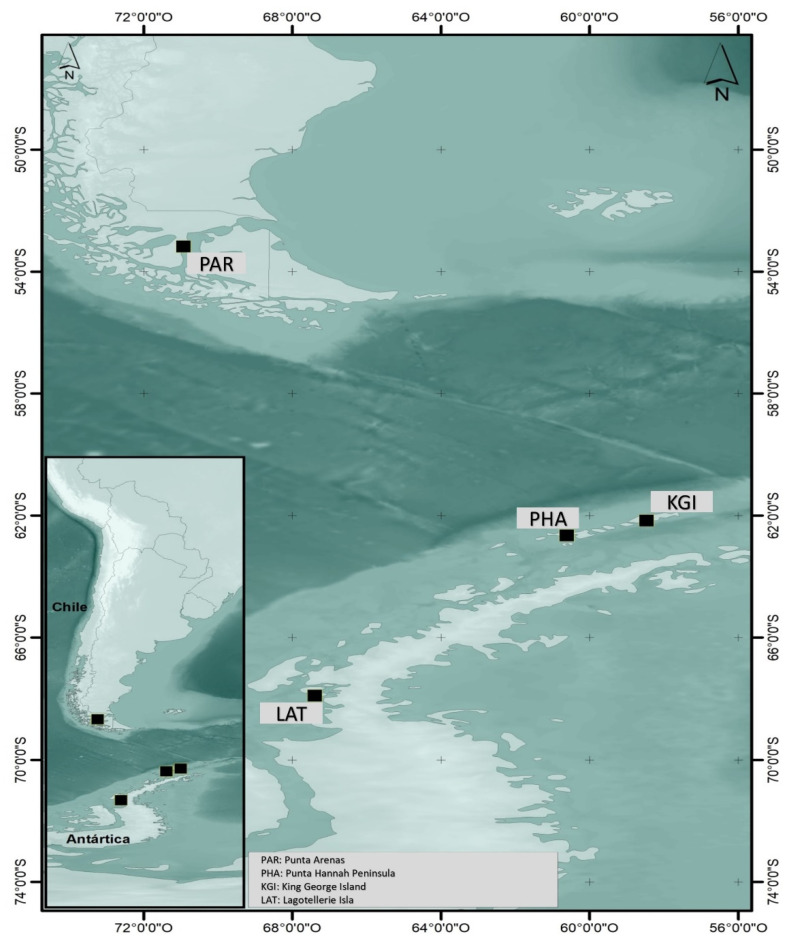
Geographical location of the four zones in which samples were taken of *Colobanthus quitensis* plants and their soils of origin.

The samples of *Colobanthus quitensis* and their respective soils of origin were collected at four sites in the far south of mainland Chile and the Antarctic territory (Figure 5). Access to the sampling sites in Antarctic territory was limited to disembarkation from the expedition vessel and as permitted by the Chilean navy. The permitted geographical zones were as follows:

- Punta Arenas (PAR) 53°10′01″ S–70°56′01″ W, the northernmost end of the gradient. This site is on mainland Chile, close to Punta Arenas airport, with low human activity.
- King George Island Station (KGI) 62°09′41″ S–58°28′10″ W, a site presenting quite strong human influence within several bases that support various scientific investigations.
- Hannah Point Peninsula (PHA) 62°39′ S–60°37′ W. This is a bay where tourists from cruise ships come ashore. There is a wide variety of fauna, with different species of penguins, terns, and petrels. *Colobanthus quitensis* is abundant, but people familiar with the site are becoming increasingly scarce.
- Lagotellerie Island (LAT) 67°53′16″ S–67°24′2″ W. This relatively pristine Antarctic island was the southernmost point of the transect. It has suffered little intervention and has scarce fauna but relatively abundant flora (*Colobanthus quitensis* and *Dechampsia antarctica*).

### 4.2. Sample Collection

At each sampling point, we manually collected three plants randomly, and three soil samples during summer 2016 (January). It should be noted that, according to scientists who regularly attend Antarctica, the abundance of *C. quitensis* was lower in that summer.

Samples of the aerial part (leaves) and roots of each sample plant were collected. Soil samples were collected to a depth of 0–10 cm by digging test trenches and by extracting the samples with PVC materials to avoid contamination by metals. The plant and soil samples were labelled and kept in plastic containers for subsequent chemical analysis.

### 4.3. Chemical Analysis

The *Colobanthus quitensis* samples (leaves and roots) were washed with bi-distilled water in laboratory. Samples were put in nylon strainer and were washed with plastic wash bottles two times. Nonmetallic instruments were used. Then, the samples were dried in a stove at 105 °C until they were the same weight. They were then milled and homogenized, and 1.0 g of plant tissue was weighed out and calcined slowly in a porcelain crucible at 500 °C for 4 h. The crucibles were allowed to cool to room temperature, and 10 mL of nitric acid and 2 mL of bi-distilled water were added. The solutions were heated on a heating plate with constant stirring until almost dry and then were filtered on 0.45 μm filters. The filtrates were brought to a final volume of 50 mL with bi-distilled water. Blank solutions were also included [46,65,66].

Following the methodologies used by Tapia [46], the soil samples were dried at 105 °C, and a representative sample (0.5 g) was mixed with 50 mL of HF-HNO_3_ (2:1) mixture and solubilized at 120 °C until almost dry with constant stirring. The resulting solution was filtered on 0.45 μm filters, washed with bi-distilled water, and made up to 50 mL in a pre-treated volumetric flask. The analyses were conducted with their respective control solutions [44].

The quantification of the metals was carried out using a spectrophotometer of atomic absorption spectroscopy with a flame (Solaar model 969, UNICAM, Cambridge, UK). For the measurement of Cd, Cu, Mn, Ni, Pb, and Zn, an air/acetylene flame was used, and for Cr, a nitrous oxide/acetylene flame was used. The detection limits for Cd, Cu, Cr, Mn, Ni, Pb, and Zn were 0.0028, 0.0045, 0.0054, 0.0016, 0.0080, 0.0130, and 0.0033 mg/L, respectively.

### 4.4. Validation Methodology

Certified reference material was used to validate the analytical methodology. BIMEP-432 (Leylandcypress/Cupressus x Leylandii) from Wageningen Evaluating Programs for Analytical Laboratories (WEPAL) was used for the analysis of plant tissues (leaves and roots). MESS-1 (marine sediment), from the National Research Council, Canada, Division of Chemistry, was used for the soil sample analysis. It should be noted that marine sediment is not the same as terrestrial sediment; however, in previous analyses, this material was successful in validating our methodology (comm. pers. J. Tapia).

### 4.5. Statistical Analysis

Our statistical analyses consisted of verifying the normality, independence, and homoscedasticity of the collected data. One-way analysis of variance was carried out using the sampling stations as the variable, and then, an a posteriori analysis was carried out, using Tukey’s test with a significance level of 95%. The patterns of the mean metal concentrations in leaves, roots, and soils in the four sampling stations were assessed via principal component analysis (PCA) with the metal data normalized. PCA was performed using the software PRIMER v.6 [67].

## 5. Conclusions

We measured Mn, Cu, Cr, Cd, Ni, Pb, and Zn in the soil, root, and leaf samples of *Colobanthus quitensis* at all of the sampling stations. We recorded the highest mean concentrations at the site with the greatest human activity, whereas the lowest mean concentrations of all of the metals were in the southernmost point from which we took the samples, a pristine island with little human intervention, scarce fauna, and abundant flora (*Colobanthus quitensis* and *Deschampsia antarctica*). Thus, the values of the metal concentrations obtained in this study, especially those recorded at Lagotellerie Island station can be taken as reference values for comparisons in future studies.

The mean concentrations of all of the metals in the soil were within the ranges reported by other authors and below the maxima permitted by the Canadian, European Union, and World Range for non-polluted soils standards. Only the Cd and Cu average concentration values were within the recommendations of the WRS. The order of the mean concentrations of the metals in soils was Mn > Zn > Cu > Cr > Pb > Ni > Cd. The concentrations of the metals studied in the root and leaf samples of *Colobanthus quitensis* were within the ranges recorded in other investigations. However, *Colobanthus quitensis* survives in the harsh environmental conditions of Antarctica, and the fact that vascular plants tolerate higher soil concentrations of MTEs better than other types must be considered. The mean concentrations of the metals in the root and leaf samples were similar, following the order Mn > Zn > Cu > Ni > Pb > Cr > Cd. If human activities continue increasing in the Antarctica, for example, the arrival of tourists in summer or researchers at scientific bases, MTE contamination could probably increase in the future in soils and plants from the study area. Our study shows that there are still areas with high human intervention and with high concentrations of MTEs, suggesting that human influence may threaten plants in Antarctica. As the MTE concentration in *C. quitensis* was influenced by proximity to human influence, we suggest that future studies must continue to monitor this Antarctic plant as MTE bioindicators or sentinel species, including a future metal pollution index.

## Data Availability

Not applicable.

## Appendix A

**Table A1:** Concentrations of Cd, Cu, Cr, Mn, Ni, Pb, and Zn (mg/kg ± S.D. in dry weight) in certified reference material (BIMEP-432) from the Wageningen Evaluating Programs for Analytical Laboratories (WEPAL).

Element	Certified Concentration (MAD)	Observed Concentration (n = 3)	Rel. Error %	Recovery (%)
Cd	1.30 (0.90) n =4	1.19 ± 0.02	−8.5	91.5
Cu	6.05 (3.50) n =6	6.47 ± 0.35	6.9	106.9
Cr	2.35 (1.65) n =5	2.16 ± 0.06	−8.1	91.9
Mn	20.0 (4.0) n =5	20.58 ± 0.91	2.9	102.9
Ni	1.15 (0.5) n =4	1.10 ± 0.09	−4.3	95.7
Pb	1.79 (1.1) n =3	1.93 ± 0.05	7.8	107.8
Zn	18.5 (2.8) n =6	19.10 ± 0.08	3.2	103.2

MAD: Median of Absolute Deviation.

**Table A2:** Concentrations of Cd, Cu, Cr, Mn, Ni, Pb, and Zn (mg/kg ± S.D. in dry weight) in certified reference material (MESS-1) from the National Research Council Canada (NRC).

Element	Certified Concentration	Observed Concentration (n = 2)	Rel. Error %	Recovery (%)
Cd	0.50 ± 0.10	0.62 ± 0.06	5.1	105.1
Cu	25.1 ± 3.8	26.1 ± 0.15	4	104
Cr	71.0 ± 11	70.0 ± 0.19	−1.4	98.6
Mn	513 ± 25	499.5 ± 3.35	−2.6	97.4
Ni	29.5 ± 2.7	30.0 ± 0.15	1.7	101.7
Pb	34.0 ± 6.1	35.1 ± 0.07	3.2	103.2
Zn	191 ± 17	198.1 ± 4.10	3.7	103.7

## Appendix B

**Table A3:** Concentrations of Cr, Cd, Cu, Pb, Ni, Mn, and Zn (mg/kg ± S.D. in dry weight “DW”) (n = 3) in the soils of the species *Colobanthus quitensis* collected at the LAT, PHA, KGI and PAR stations with the mean concentrations of each metal.

Stations	LAT	PHA	KGI	PAR	Mean
Cr	8.73 ± 0.02	31.78 ± 1.44	40.17 ± 3.11	20.98 ± 1.75	25.4 ± 11.8
Cd	1.27 ± 0.13	0.72 ± 0.18	1.89 ± 0.13	1.98 ± 0.20	1.5 ± 0.5
Cu	18.82 ± 1.47	18.59 ± 0.44	104.80 ± 7.32	41.37 ± 3.27	45.9 ± 35.2
Pb	1.87 ± 0.00	6.26 ± 1.88	37.30 ± 2.56	24.68 ± 1.77	17.5 ± 14.3
Ni	6.95 ± 1.38	16.74 ± 1.08	23.48 ± 4.42	13.59 ± 1.16	15.2 ± 5.9
Mn	397.29 ± 10.87	649.27 ± 19.16	833.39 ± 23.48	451.79 ± 21.31	582.9 ± 172.3
Zn	56.80 ± 2.17	59.64 ± 2.31	84.80 ± 6.26	78.58 ± 3.78	70.0 ± 12.0

**Table A4:** Concentrations of Cr, Cd, Cu, Pb, Ni, Mn, and Zn (mg/kg ± S.D. in dry weight “DW”) (n = 3) in the roots of the species *Colobanthus quitensis* collected at the LAT, PHA, KGI and PAR stations with the mean concentrations of each metal.

Stations	LAT	PHA	KGI	PAR	Mean
Cr	1.42 ± 0.17	3.77 ± 0.70	5.62 ± 0.15	2.28 ± 0.50	3.30 ± 1.60
Cd	2.56 ± 0.13	2.02 ± 0.35	1.04 ± 0.20	0.47 ± 0.16	1.50 ± 0.80
Cu	10.34 ± 1.14	9.48 ± 0.37	50.71 ± 1.94	13.22 ± 0.08	20.90 ± 17.20
Pb	3.91 ± 0.36	2.68 ± 0.58	5.25 ± 0.56	1.72 ± 0.92	3.40 ± 1.30
Ni	8.71 ± 0.60	10.18 ± 0.13	9.69 ± 1.23	2.59 ± 0.26	7.80 ± 3.00
Mn	125.67 ± 2.90	381.92 ± 7.96	383.19 ± 13.50	481.99 ± 15.38	343.20 ± 131.90

**Table A5:** Concentrations of Cr, Cd, Cu, Pb, Ni, Mn, and Zn (mg/kg ± S.D. in dry weight) (n = 3) in the leaves of the species *Colobanthus quitensis* collected at the LAT, PHA, KGI, and PAR stations with the mean concentrations of each metal.

Stations	LAT	PHA	KGI	PAR	Mean
Cr	0.80 ± 0.06	2.61 ± 0.19	4.41 ± 0.16	1.48 ± 0.03	2.30 ± 1.40
Cd	0.15 ± 0.05	0.13 ± 0.02	0.23 ± 0.06	0.19 ± 0.00	0.20 ± 0.04
Cu	6.17 ± 0.41	11.70 ± 0.59	36.60 ± 4.80	8.58 ± 0.71	15.80 ± 12.20
Pb	2.33 ± 0.29	4.02 ± 0.23	6.46 ± 0.11	4.88 ± 1.25	4.40 ± 1.50
Ni	6.49 ± 0.62	7.13 ± 0.08	7.65 ± 0.49	3.55 ± 0.91	6.20 ± 1.6
Mn	81.94 ± 3.53	374.92 ± 18.57	326.61 ± 10.64	243.51 ± 6.39	256.70 ± 111.3
Zn	33.98 ± 1.27	44.95 ± 2.73	59.84 ± 0.46	40.18 ± 4.57	44.70 ± 9.60
